# Topological Schemas of Memory Spaces

**DOI:** 10.3389/fncom.2018.00027

**Published:** 2018-04-24

**Authors:** Andrey Babichev, Yuri A. Dabaghian

**Affiliations:** ^1^Department of Computational and Applied Mathematics, Rice University, Houston, TX, United States; ^2^Department of Neurology, The University of Texas McGovern Medical School, Houston, TX, United States

**Keywords:** place cells, hippocampus, cell assemblies, memory space, cognitive map, topology

## Abstract

Hippocampal cognitive map—a neuronal representation of the spatial environment—is widely discussed in the computational neuroscience literature for decades. However, more recent studies point out that hippocampus plays a major role in producing yet another cognitive framework—the memory space—that incorporates not only spatial, but also non-spatial memories. Unlike the cognitive maps, the memory spaces, broadly understood as “networks of interconnections among the representations of events,” have not yet been studied from a theoretical perspective. Here we propose a mathematical approach that allows modeling memory spaces constructively, as epiphenomena of neuronal spiking activity and thus to interlink several important notions of cognitive neurophysiology. First, we suggest that memory spaces have a topological nature—a hypothesis that allows treating both spatial and non-spatial aspects of hippocampal function on equal footing. We then model the hippocampal memory spaces in different environments and demonstrate that the resulting constructions naturally incorporate the corresponding cognitive maps and provide a wider context for interpreting spatial information. Lastly, we propose a formal description of the memory consolidation process that connects memory spaces to the Morris' cognitive schemas-heuristic representations of the acquired memories, used to explain the dynamics of learning and memory consolidation in a given environment. The proposed approach allows evaluating these constructs as the most compact representations of the memory space's structure.

## 1. Introduction

In the neurophysiological literature, the functions of mammalian hippocampus are usually discussed from the following two main perspectives. One group of studies addresses the role of the hippocampus in representing the ambient space in a cognitive map (Tolman, 1948; Moser et al., 2008), and the other focuses on its role in processing non-spatial memories, notably the episodic memory frameworks (Eichenbaum, 2004; Dere et al., 2006; Hassabis et al., 2007; Crystal, 2009). Active studies of the former began with the discovery of the “place cells”—hippocampal neurons that fire action potentials in discrete regions of the environment—their respective “place fields.” It was demonstrated, e.g., that place cell firing can be used to reconstruct the animal's trajectory on moment by moment basis (Jensen and Lisman, 2000; Barbieri et al., 2005; Guger et al., 2011), or to describe its past navigational experiences (Carr et al., 2011) and even its future planned routs (Dragoi and Tonegawa, 2013), which suggests that the cognitive map encoded by the hippocampal network provides a foundation of the animal's spatial memory and spatial awareness (O'Keefe and Nadel, 1978; Best et al., 2001).

**Figure 1:**
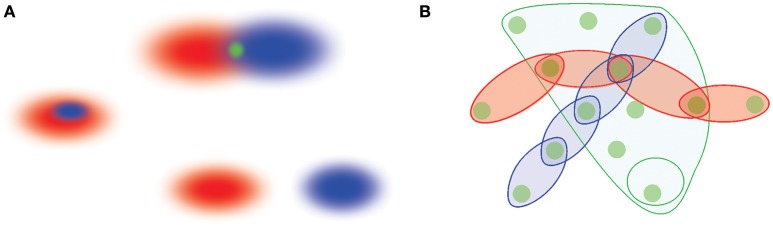
A schematic illustration of memory space concept. **(A)** Memory elements are viewed as regions in memory space, *r*_1_ and *r*_2_ (red and blue ovals). The overlapping regions yield a smaller region in the intersection that represents a shared memory (top figure). Alternatively, one memory region can also contain another (the middle figure), or two memory regions can be separate from one another (bottom figure). **(B)** Memory elements jointly form a cohesive framework—the memory space—into which different memory sequences are embedded. The episodes connected in sequences can be viewed as chains of interconnected regions that run across the memory space, whereas memories that are “broader in the features” are represented by extended, space-like domains of the memory space. The most elementary, indecomposable elements shared between distinct behavioral episodes represent “nodes”—the elementary locations in the memory space.

On the other hand, it was observed that hippocampal lesions result in severe disparity in episodic memory function, i.e., the ability to produce a specific memory episode and to place it into a context of preceding and succeeding events. In healthy animals, episodic sequences consistently interleave with one another, yielding an integrated, cohesive semantic structure (Wallenstein et al., 1998; Agster et al., 2002; Fortin at al, 2002, 2004; MacDonald et al., 2011). In Eichenbaum et al. (1999) and Eichenbaum (2000, 2015) it was therefore suggested that the overall memory framework should be viewed as an abstract “memory space” M, in which individual memories correspond to broadly understood “locations” or “regions.” The relationships between memories are represented via spatial relationships between these regions, such as adjacency, overlap or containment (Figure 1). It was also suggested that the animals can “conceptually navigate” the memory space by perusing through learned associations, i.e., by comparing and contrasting directly connected memories and inferring relationships between indirectly linked ones (Buzsaki and Moser, 2013; Buffalo, 2015). In this approach, the conventional spatial inferences that enable spatial navigation of physical environments based on cognitive maps are viewed as particular examples of navigating a memory space, which in general allows inferring associations and producing reasoning chains of abstract nature (Eichenbaum et al., 1999). In other words, the concept of memory space generalizes the notion of cognitive map: the latter unifies specifically spatial memories and hence forms a substructure or a subspace embedded into a larger memory space.

### 1.1. Extended topological hypothesis

Traditionally, the cognitive map is viewed as a Cartesian map of animal's locations, distances to landmarks, angles between spatial cues and so forth (O'Keefe and Nadel, 1978; Best et al., 2001). However, increasing amount of experimental evidence suggests that this map is based on representing qualitative spatial relationships rather than precise spatial metrics. For example, it has been demonstrated that if the environment gradually changes its shape in a way that preserves relative order of spatial cues, then the temporal order of the place cell spiking and the relative arrangement of the place fields remain invariant throughout the change (Muller and Kubie, 1987; Gothard et al., 1996; Lever et al., 2002; Leutgeb et al., 2005; Wills et al., 2005; Diba and Buzsaki, 2008; Colgin et al., 2010; Wu and Foster, 2014). This suggests that place cell coactivities emphasize contiguities between locations as well as the temporal sequence in which they are experienced, and hence that the hippocampus encodes a flexible framework of spatial relationships—a topological map of space (Poucet, 1993; Wallenstein et al., 1998; Alvernhe et al., 2012; Dabaghian et al., 2014).

The mathematical nature of memory space has not been addressed in computational neuroscience literature. However, general properties of the episodic memory frameworks suggest that such a space should also be viewed as primarily topological. Indeed, the “regions” or “locations” in M are abstract concepts that are not attributed any particular geometric features, such as shape or size, and the relationships between these regions do not involve precise metric calculations of distances and angles. Rather, the memory space is based on qualitative spatiotemporal relationships, which is a defining property of topological spaces (Vickers, 1989). Thus, the topological perspective provides a common ground for both “spatial” and “non-spatial” aspects of the hippocampal functions. In fact, the contraposition between these two specialties of the hippocampus might have originated, in the first place, from an excessive “geometrization” of the cognitive map. If the hippocampal spatial map is Cartesian, then it is not entirely clear which mechanism could be responsible for representing coordinates, distances, angles, etc., in the spatial domain and only qualitative relationships between memory items in the mnemonic domain. On the other hand, it is hard to attribute geometric characteristics to the elements of the memory space, especially to the non-spatial memories, and it is unclear what role geometry would play in that space. However, if both the cognitive map and the memory space are viewed as topological, based on relational representation of information, then the principles of spatial representation and mnemonic memory functions converge (Dabaghian et al., 2014). Taken together, these arguments suggest that the hippocampal network encodes a generic topological framework, which may be manifested as a cognitive map or as a more general memory space, depending on the context and the nature of the encoded information.

In the following, we propose a theoretical framework that incorporates both the cognitive maps and the memory spaces and allows modeling them constructively, as epiphenomena of neuronal activity. In particular, it allows relating the topological properties of the memory space to the parameters of the place cell spiking, e.g., to the rate and the spatial selectivity of firing. The proposed approach also allows connecting the concept of memory space to the Morris' cognitive schemas—abstract, heuristic representations of acquired knowledge, skills and memories, used to explain the dynamics of learning and memory consolidation (Tse et al., 2007; Wang and Morris, 2010). In our approach, these constructions emerge as the most compact representations of the memory space's structure and can be evaluated from the spiking data.

## 2. The model

In Babichev et al. (2016a) we proposed theoretical approach for modeling cognitive maps, which allows combining the information provided by the individual place cells into a large-scale topological representation of the environment. Following the standard neurophysiological paradigm, the model assumes, firstly, that the activity of each individual place cell *c*_*k*_ encodes a spatial region *r*_*k*_ that serves as a building block of the cognitive map. Secondly, it assumes that the large-scale structure of the cognitive map emerges from the connections between these regions, encoded in a population place cell assemblies—functionally interconnected groups that synaptically drive their respective reader-classifier (readout) neurons in the downstream networks (Harris et al., 2003; Buzsaki, 2010). A particular readout neuron integrates the presynaptic inputs and produces a series of spikes, thus actualizing a specific relationship ρ(*r*_1_, *r*_2_, …, *r*_*m*_) between the regions *r*_1_, *r*_1_,…*r*_*m*_.

**Figure 2:**
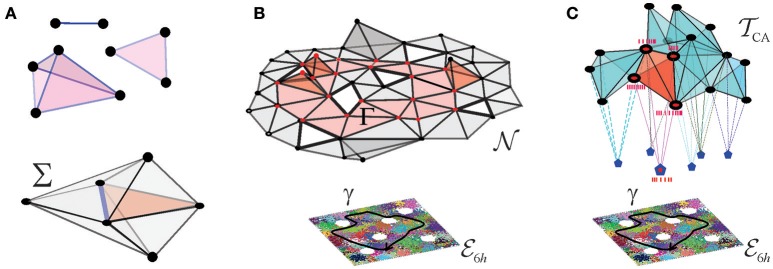
Coactivity complex and the cell assembly complex. **(A)** Three exemplary simplexes: a one-dimensional (1*D*) link, a 2*D* triangle, and a 3*D* tetrahedron are shown on the top. Together a few simplexes form a small simplicial complex Σ shown below. Note that the 2*D* and 3*D* simplexes surrounding a 1*D* simplex (the blue link) form its vicinity–this observation will be used in the Alexandrov space construction. **(B)** The nerve complex N represents the pattern of overlaps between place fields covering a given environment, every simplex σ∈N represents a combination σ = [π_*i*_0__, π_*i*_1__, …, π_*i*_*d*__] of overlapping place fields, π_*i*_0__ ∩ π_*i*_1__ … ∩ π_*i*_*d*__ ≠ ∅. The bottom of the panel shows place field map, M(E) of a square environment with six holes, $E_{6h}$, traversed by a trajectory γ (black line). Place cells are shown as vertices of the simplexes: the active place cells are shown as red points and the inactive ones as black points. The figure schematically represents a 2*D*-skeleton of T, used to compute the topological features of the underlying environment. The simplexes representing place cell combinations that become coactive as the animal navigates along γ form a simplicial path Γ, shown in red. The simplicial path encircles the hole in the coactivity complex that represents the physical hole in the environment. The coactivity complex T is an implementation of the nerve complex in temporal domain: every simplex, σ∈T represents a combination of coactive place cells, σ = [*c*_1_, *c*_2_, …, *c*_*n*_]. Over time, T becomes structurally identical to N. **(C)** Simplexes of the cell assembly complex $T_{CA}$ represent the cell assemblies, shown as interconnected cliques of vertexes—that jointly drive readout neurons in the downstream networks (shown as pentagons to which place cells connect synaptically). Red clique represents an ignited place cell assembly, eliciting a spiking response from its readout neuron.

A few schematic models were built in Dabaghian et al. (2012), Arai et al. (2014), Basso et al. (2016), Hoffman et al. (2016), and Babichev et al. (2016a,b) based on the observation that an assembly of place cells *c*_1_, *c*_2_, …, *c*_*m*_, can be formally represented by an “abstract simplex,” σ = [*c*_1_, *c*_2_, …, *c*_*m*_]. In mathematics, the term “simplex” usually designates a convex hull of (*d* + 1) points in a space of at least *d* dimensions. For example, a first order simplex can be visualized as a zero dimensional point, a second order simplex—as a line segment with a vertex at each end, a third order simplex—as a triangle with three vertices, etc. (Figure 2A). However, in topological applications that address the net, large-scale properties of aggregations of simplexes—simplicial complexes—the shapes of the simplexes play no role: the information is contained only in the combinatorics of the vertexes shared by the adjacent simplexes. This motivates using the so-called “abstract simplexes”—combinatorial abstractions, defined without any reference to geometry, simply as sets of (*d* + 1) elements of arbitrary nature. Thus, abstract simplexes and simplicial complexes retain only one basic property of their geometric counterparts: just as the triangles or the tetrahedra include their facets, an abstract simplex of order (*d* + 1) includes all its subsimplexes of lower orders. As a consequence, a non-empty overlap of a pair of simplexes σ and σ′ is a subsimplex of both σ and σ′ (Figure 2A).

Previous studies (Curto and Itskov, 2008; Chen et al., 2012; Dabaghian et al., 2012; Arai et al., 2014; Babichev et al., 2016b; Basso et al., 2016; Hoffman et al., 2016) suggest that the topological theory of simplicial complexes provides a remarkably efficient semantics for describing many familiar concepts and phenomena of hippocampal physiology, as outlined in the following examples.

### 2.1. Example 1. a nerve complex N

A set of overlapping place fields, π_*i*_0__ ∩ π_*i*_1__ ∩ … ∩ π_*i*_*d*__ ≠ ∅ produced by the place cells *c*_*i*_0__, *c*_*i*_1__, …*c*_*i*_*d*__ can be represented by an abstract simplex σ = [π_*i*_0__, π_*i*_1__, …, π_*i*_*d*__]. The totality of all such simplexes produced for a given place field map $M_{E}$ then forms a simplicial complex—the nerve of the cover $N\left(M_{E}\right)$ (Curto and Itskov, 2008; Chen et al., 2012; Dabaghian et al., 2012). Every individual place field corresponds to a vertex, σ_*i*_, of $N\left(M_{E}\right)$; each non-empty overlap of two place fields, π_*i*_ ∩ π_*j*_ ≠ ∅, contributes a link $\sigma_{ij}\in N\left(M_{E}\right)$, a non-empty overlap of three place fields, π_*i*_ ∩ π_*i*_ ∩ π_*k*_ ≠ ∅, contributes a facet $\sigma_{ijk}\in N\left(M_{E}\right)$, and so forth. The Alexandrov-Čech theorem (Alexandroff, 1928; Čech, 1932) states that if the overlapping regions are contractible in E (i.e., can be continuously retracted into a point), then $N\left(M_{E}\right)$ and E have the same number of holes, loops and handles in different dimensions—mathematically, they have the same homologies, $H_{*}\left(N\left(M_{E}\right)\right)=H_{*}\left(E\right)$. Thus, the nerve complex may serve as a schematic representation of the topological information contained in the place field map $M_{E}$ (Babichev et al., 2016a).

### 2.2. Example 2. the coactivity complex T

In the brain, the information is represented via temporal relationships between spike trains, rather than artificial geometric constructs such as place fields. However, the place cell spiking patterns can also be described in terms of a simplicial “coactivity” complex $T\left(M_{E}\right)$, which may be viewed as an implementation of the nerve complex $N\left(M_{E}\right)$ in the temporal domain. In this construction, every active place cell *c*_*i*_ is represented by a vertex, σ_*i*_, of $T\left(M_{E}\right)$; each coactive pair of cells, *c*_*i*_ and *c*_*j*_, contributes a link $\sigma_{ij}=\left[c_{i},c_{j}\right]\in T\left(M_{E}\right)$, a triplet of coactive cells contributes a facet $\sigma_{ijk}=\left[c_{i},c_{j},c_{k}\right]\in T\left(M_{E}\right)$, and so forth. As a whole, the coactivity complex T represents the entire pool of the coactive place cell combinations. Numerical simulations carried out in Dabaghian et al. (2012), Arai et al. (2014), Basso et al. (2016), and Hoffman et al. (2016) demonstrate that if the parameters of place cells' spiking fall into the biological range, then $T\left(M_{E}\right)$ faithfully represents the topology of two- and three-dimensional environments and serves as a schematic representation of the information provided by place cell coactivity (Figure 2B).

### 2.3. Example 3. cell assembly complex $T_{CA}$

Physiologically, not all combinations of coactive place cells are detected and processed by the downstream networks. Therefore, in order to describe only the physiologically relevant coactivities, one can construct a smaller “cell assembly complex” $T_{CA}\left(M_{E}\right)$, whose maximal simplexes represent the actual cell assemblies, rather than arbitrary combinations of coactive cells (Figure 2C). Such a complex can then play two complementary roles: first, it can schematically represent the architecture of the cell assembly network (i.e., define explicitly which cells group into which assemblies) and second, it can represent the information encoded by this network and hence serve as a schematic model of the cognitive map (Babichev et al., 2016b).

Previous studies (Dabaghian et al., 2012; Arai et al., 2014; Basso et al., 2016; Hoffman et al., 2016) concentrated on the lower dimensions (*D* ≤ 3) of the coactivity and of cell assembly complexes used to represent spatial information, whereas the higher dimensions were not addressed or physiologically interpreted. However, a schematic representation of both spatial and non-spatial memories should include the full scope of relationships encoded by the cell assemblies; we will therefore use the full coactivity complex $T_{CA}\left(M_{E}\right)$ to model a multidimensional memory space.

### 2.4. A constructive approach to topology and continuity

We now make a short mathematical digression to outline the key notions necessary for discussing the topology of memory spaces. In general, defining a topological space requires two constituents: a set *X* of spatial primitives—the “building blocks of space,” and a set of relationships between them, which define spatial order and spatial connectivity. In the standard approach, the topological spaces are comprised of an infinite amount of infinitesimal points, and a framework of proximity and remoteness relationships emerges as a matter of combining these points into “topological neighborhoods” (see section 4). Such system of neighborhoods is referred to as a topology on *X*, which we will denote as Ω(*X*). In order for the neighborhoods to be mutually consistent, it is required that their unions and finite intersections should also be neighborhoods from Ω(*X*) (so-called Hausdorff axioms, see section 4). Once a consistent framework of neighborhoods is defined, the elements of the set *X* can be viewed as “spatial locations” and the set *X* itself—as a topological space. For example, the environment E, viewed as a domain of Euclidean space, contains a continuum of infinitesimal points with Cartesian coordinates (*x, y*). The standard selection of topological neighborhoods in this case is the set of open balls of rational radii, centered at the rational points, and their combinations. This is the conventional Euclidean topology $\Omega_{E}\left(E\right)$ used in calculus and in standard geometries (Alexandrov, 1965).

Modeling a “memory space” requires modifying this approach in two major aspects. First, since a memory space emerges from the spiking activity of a finite number of neurons, it must be modeled as *finite topological space* (Alexandroff, 1937; McCord, 1966; Stong, 1966), i.e., as a space that may contain only a finite number of elementary locations. Second, since every location is encoded by a finite ensemble of place cells, each one of which represents an extended region, the “spatial primitives” in memory space must be finite domains, rather than infinitesimal points. The latter approach underlies the so-called pointfree (or “pointless”) topologies, geometries (Laguna, 1922; Weil, 1938; Johnstone, 1983; Roeper, 1997; Sambin, 2003), and mereotopologies (Cohn and Hazarika, 2001; Cohn and Varzi, 2003), in which finite regions are considered as the primary objects, whereas the points appear as secondary abstractions. As discussed below, these approaches provide suitable frameworks for modeling the biological mechanisms of spatial information processing.

### 2.5. A simplicial schema of a memory space

To build a model of a memory space, we start by noticing that simplicial complexes themselves may be viewed as topological spaces, because the relationships between simplexes in a simplicial complex Σ naturally define a set of topological proximity neighborhoods. Indeed, a neighborhood of a simplex σ is formed by a collection of simplexes that include σ (Figure 2A). It can be verified that the unions and the intersections of so-defined neighborhoods satisfy the Hausdorff axioms and hence that any simplicial complex Σ may be viewed as a finitary topological space A(Σ) (see section 4). In mathematical literature, such spaces are referred to as Alexandrov spaces, after their discoverer, P. S. Alexandrov (Alexandroff, 1937), which motivates our notation.

Importantly, the construction of Alexandrov spaces applies to “abstract” simplicial complexes, whose simplexes may represent collections of elements of arbitrary nature and hence possess a great contextual flexibility. In our model, individual simplexes represent combinations of coactive place cells, believed to encode memory episodes. We may therefore view the pool of coactive neuronal combinations as a topological space from two perspectives. On the one hand, one can consider a formal “space of coactivities” $A_{E}\left(T_{CA}\right)$ defined, as the corresponding coactivity complexes, in terms of the neuronal spiking parameters. On the other hand, assuming that the combinatorial relationships between groups of coactive cells capture relationships between the corresponding memory episodes, one may view the collection of memories *represented* by these neuronal activity patterns as elements of a topological *memory space* $M_{E}\left(T_{CA}\right)$. In other words, one can view the Alexandrov space $A_{E}\left(T_{CA}\right)$ as a model of the memory space $M_{E}\left(T_{CA}\right)$ induced by the corresponding cell assembly network. In particular, such model can be used to connect the physiological parameters of the latter and the topological characteristics of $M_{E}\left(T_{CA}\right)$, as we discuss below. Since all subsequent analyses are carried out only for the memory spaces induced from cell assembly complexes, we will suppress the reference to $T_{CA}$ in the memory space notation.

We would like to note here, that since the simplexes are not structureless objects (e.g., one combination of coactive cells represented by simplex σ_1_ may overlap with another combination, represented by a simplex σ_2_, yielding a third combination/simplex σ_3_), they represent extended regions, rather than structureless points. As a result, the memory space $M_{E}$ naturally emerges as a region-based, or “pointfree” space, in which individual memory episodes correspond to finite regions. Nevertheless, one can easily construct a conventional, i.e., point-based, topological space in which a finite set of elementary locations—the “points”—is organized into the same system of proximity neighborhoods as its region-based counterpart (see section 4). In this construction, the “elementary locations” are simply the smallest regions of $M_{E}$, i.e., the ones that cannot be further subdivided using the information contained in the place cell coactivity—the “nodes of the memory space,” in terminology of Eichenbaum et al. (1999). In the spatial context, they correspond to the atomic, indecomposable regions. For example, a mini-memory space encoded by two place cells may contain three “atomic” regions: e.g., the region marked by the activity of first, but not the second cell, the region marked by the coactivity of both cells and the region marked by the activity of the second, but not the first cell (Figure 1A and Figure 12.1 in Munkres, 2000). In the following we will discuss the organization of such regions in order to establish important properties of the memory spaces, e.g., a continuous mapping of the environment E into a memory space $M_{E}$.

## 3. Results

### 3.1. Continuity in memory space

The discrete memories that comprise a memory space may be triggered by constellations of cues and/or actions, that drive the activity of a particular population of cell assemblies (Buzsaki et al., 2014). Activation of one cell assembly may excite adjacent cell assemblies that represent overlapping memory elements. Thus, as the animal navigates the environment, the cell assemblies ignited along a path γ form an “activity packet” that moves across the network (Samsonovich and McNaughton, 1997; Touretzky et al., 2005; Romani and Tsodyks, 2010). If the cell assembly network is represented by a complex $T_{CA}$, this packet is represented by a group of “active” simplexes that moves across $T_{CA}$, tracing a simplicial path Γ (Figure 2B). As discussed in Dabaghian et al. (2012), Arai et al. (2014), Basso et al. (2016), Dabaghian (2016), and Hoffman et al. (2016), the structure of the simplicial paths captures the shape of the corresponding physical paths and hence represents the connectivity of the environment. For example, a contractible simplicial path corresponds to a contractible physical rout, whereas a non-contractible simplicial path marks a non-traversable domain occupied by an obstacle, e.g., by a physical obstruction or by a predator (Figures 2B,C).

Intuitively, one would expect that a continuous physical trajectory should be represented by a “continuous succession” of activity regimes of the place cells that represents a continuous sequence of memory episodes. Indeed, the topological structure of the memory space provides a concrete meaning for this intuition. It can be shown that the environment E maps continuously into the memory space $M_{E}$, and in particular, that each continuous trajectory γ traced by the animal in the physical environment maps into a continuous path ℘ in the memory space $M_{E}$ (see section 4). It should be noted however, that these are different continuities: the physical trajectory γ is continuous in the Euclidean topology of the environment, whereas the path ℘ is continuous in the topology of the memory space. This distinction is due to fact that the environment E and the memory spaces $M_{E}$ are not topologically equivalent to each other: one can map the rich Euclidean topology onto the discrete finite topology of a memory space, but not vice versa. In other words, despite the continuity of mapping from E into $M_{E}$, the memory space remains only a discretization of the environment, which nevertheless serves as a topological representation of E and can be continuously navigated.

### 3.2. Topological properties of memory spaces

Topological properties of memory spaces can be studied from two perspectives: from the perspective of algebraic topology that captures the large-scale structure of $M_{E}$ in terms of topological invariants (Munkres, 2000), or from the perspective of the so-called general topology (Alexandrov, 1965), which describes the topological “fabric” of $M_{E}$, in terms of the proximity neighborhoods.

The algebraic-topological properties of the coactivity complexes were studied in Dabaghian et al. (2012), Babichev et al. (2016b), and Babichev and Dabaghian (2017a,b). There it was demonstrated that if place cell populations operate within biological parameters, then the number of topological loops in different dimensions of the coactivity complex—the Betti numbers $b_{n}\left(T_{CA}\right)$ (Munkres, 2000)—match the Betti numbers of the environment $b_{n}\left(E\right)$. Moreover, the correct shape of the coactivity complex emerges within a biologically plausible period that was referred to as learning time, *T*_min_. These results apply directly to the memory spaces, since the Betti numbers of a memory space $M_{E}$ are identical to those of the coactivity complex $T_{CA}$ that produced it (Alexandroff, 1937). (For a mathematically oriented reader, we mention that the homological structure of $M_{E}$ should be defined in terms of singular homologies, whereas the structure of the coactivity complex is described in terms of simplicial homologies. However, for the cases considered below, these homologies coincide, so we omit the discussion of the differences, McCord, 1966). This implies, in particular, that the memory space that correctly represents the topology of the environment emerges together with the corresponding coactivity complex during the same learning time *T*_min_, for the same set of spiking parameters (in terminology of Dabaghian et al. (2012), within the “learning region,” L).

Importantly, the learning times and other global characteristics of $T_{CA}$ produced via algebraic topology techniques are insensitive to many details of the place cell spiking activity (Dabaghian et al., 2012; Babichev et al., 2016b; Babichev and Dabaghian, 2017a,b). For example, the learning time *T*_min_ depends mostly on the mean place field sizes and the mean peak firing rates, but it does not depend strongly on the spatial layout of the place fields or on the limited spiking variations. The question arises, how sensitive is the “fabric” of the memory space to the parameters of neuronal activity?

**Figure 3:**
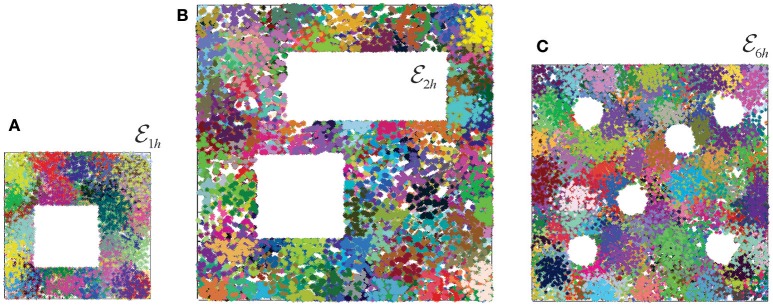
Place field maps in three simulated environments. **(A)** An example of a place field map simulated in a 1 × 1 m environment with one hole in the middle, $E_{1h}$, that was previously studied in Dabaghian et al. (2012), Basso et al. (2016), and Babichev et al. (2016b). Dots of different colors represent spikes produced by different place cells. Clusters of dots represent the corresponding place fields. **(B)** A place field map simulated in 2 × 2 m environment with two holes, $E_{2h}$, studied in Arai et al. (2014). **(C)** The third environment $E_{6h}$ (1.6 × 1.6 m in size) is similar to the behavioral arena studied in Tse et al. (2007), where the concept of the Morris' schemas was introduced. Ten different place field maps were simulated in each environment and used to produce a cell assembly network, as described in Babichev et al. (2016b). The mean size of the place fields (20 cm) and the mean firing rate of the place cells (14 Hz) is the same in all cases.

To address this question, we simulated ten different place field maps *M*_*i*_, *i* = 1, …, 10, in three environments (Figure 3), and verified that the corresponding nerves $N_{E}\left(M_{i}\right)$, coactivity complexes $T\left(M_{i}\right)$ and cell assembly complexes $T_{CA}\left(M_{i}\right)$ produced the required large-scale topological characteristics (i.e., the same Betti numbers: $b_{0}\left(E_{1h}\right)=b_{0}\left(E_{2h}\right)=b_{0}\left(E_{6h}\right)=1$, $b_{1}\left(E_{1h}\right)=1$, $b_{1}\left(E_{2h}\right)=2$, $b_{0}\left(E_{6h}\right)=6$, and $b_{n}\left(E_{1h}\right)=b_{n}\left(E_{2h}\right)=b_{n}\left(E_{6h}\right)=0$, *n* ≥ 2). We then built and analyzed the memory spaces for the cell assembly complexes, and analyzed their general-topological structure. Mathematically, the discrete topology of an Alexandrov space can be represented by a numerical matrix—the Stong matrix $S_{A}$, which enables effective numerical analyses (see section 4 and Stong, 1966). Analyzing the Stong matrices for $M_{1h}$, $M_{2h}$, and $M_{6h}$, we observed that the memory spaces constructed for different place field maps in the same environment have different topologies. In other words, a memory space $M_{E}\left(M_{i}\right)$ encoded by a cell assembly network that corresponds to the place field map *M*_*i*_ cannot, in general, be continuously deformed into the memory space $M_{E}\left(M_{j}\right)$, that corresponds to place field map *M*_*j*_ in the same environment. From the mathematical perspective, this outcome is not surprising: since memory spaces are topologically inequivalent to the environment (a continuous mapping E→M exists but the continuous mapping M→E does not), two different memory spaces produced in the same environment may be inequivalent to each other. However, from a neurophysiological perspective, these results imply that a memory space reflects not only the large-scale topological structure of the environment, but also the specifics of a particular place field map, e.g., local spatial relationships between individual place fields.

**Figure 4:**
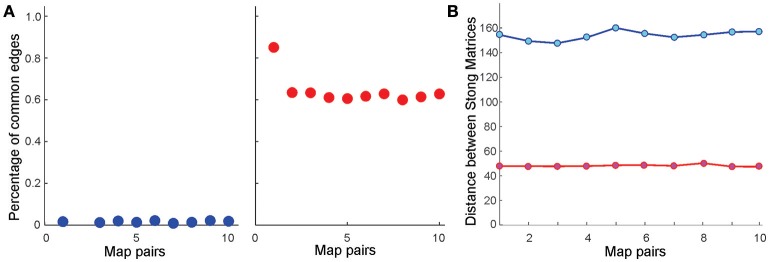
Similarity between memory spaces and place field remapping. **(A)** Proportion of one-dimensional simplexes (links) shared by ten pairs of coactivity complexes, $T_{CA}\left(M_{i}\right)$ and $T_{CA}\left(M_{j}\right)$, induced from ten pairs of place field maps in the six-hole environment $E_{6h}$. Left panel illustrates the case in which the centers of the place fields in *M*_*i*_ and *M*_*j*_ are independently scattered (global remapping); right panel illustrates the case in which the place field positions are fixed, but the place cells' firing rates and place field sizes are altered by 5% (rate remapping). In the latter case, most links are preserved, implying that the one-dimensional “skeleton” of the coactivity complex (Munkres, 2000) [or the corresponding coactivity graph G (Babichev et al., 2016a) is largely preserved in rate remappings]. **(B)** The distance norms between the Stong matrices in both global (blue) and rate (red) remappings are significant, implying that the corresponding memory spaces $M_{6h}\left(M_{i}\right)$ and $M_{6h}\left(M_{j}\right)$ are topologically distinct (see Section 4). However, the change of the memory space's topology in rate remapping is smaller than in global remapping.

Further analyses point out that even if the place field map is geometrically the same but the firing rates change by less than 5%, the cell assembly networks built according to the methods outlined in Babichev et al. (2016b) also change. As a result, the corresponding memory spaces come out to be topologically distinct from one another, although the differences between their respective Stong matrices are smaller than the differences between the Stong matrices induced by the different maps place field maps (Figure 4).

These results can be physiologically interpreted in the context of the so-called place field remapping phenomena, which we briefly outline as follows. As mentioned in the Introduction, if the changes in the environment are gradual, then the relative order of the place fields in space remains the same and place cells exhibit only small changes in the frequency of spiking (Colgin et al., 2008; Dupret et al., 2010). In contrast, if an environment is changed abruptly, e.g., if major cues suddenly appear or disappear, then the place cells may independently shift the locations of their place fields across the entire environment and significantly change their firing rates, i.e., one place field map is substituted by another (Fyhn et al., 2007; Kammerer and Leibold, 2014; Geva-Sagiv et al., 2016). The former phenomenon, known as *rate* remapping, is believed to represent variations of contextual experiences embedded into a stable spatial code, while latter, the *global* remapping, is believed to indicate a restructuring of cognitive representation of the environment. This is confirmed by our model: the differences between the memory spaces produced by two geometrically distinct place field maps *M*_*i*_ and *M*_*j*_ (physiologically, one can view a place field map *M*_*j*_ as a result of a remapping from a map *M*_*i*_) are large, whereas rate remapping produces much smaller variations in the structure of the memory space (Figure 4). In either case, the corresponding memory spaces are continuous images of the environment (i.e., a continuous mapping $E\to M_{E}$ exists in all cases) and $M_{E}$ can be continuously navigated, (see Supplementary Movies 1–3). In particular $M_{E}$ always correctly represents the large-scale topology of the environment [the Betti numbers $b_{n}\left(E\right)$ and $b_{n}\left(M_{E}\right)$ match for all *n*s].

### 3.3. Reduction of the memory spaces

Over time, the memory frameworks undergo complex changes: detailed spatial memories initially acquired by the hippocampus become coarser-grained as they consolidate into long-term memories stored in the cortex (Rosenbaum et al., 2004; Winocur and Moscovitch, 2011; Hirshhorn et al., 2012; Preston et al., 2013). From the memory space's properties perspective, this suggests that a memory space associated with a particular memory framework (e.g., with a particular environment) looses granularity but preserves its overall topological structure. The physiological mechanisms underlying these processes and the theoretical principles of memory consolidation are currently poorly understood and remain a matter of debate (O'Reilly et al., 2000; Benna and Fusi, 2016). However, the topological framework proposed above allows an impartial, schematic description of consolidating the topological details in memory spaces and producing a more compact representations of the original memory framework.

**Figure 5:**
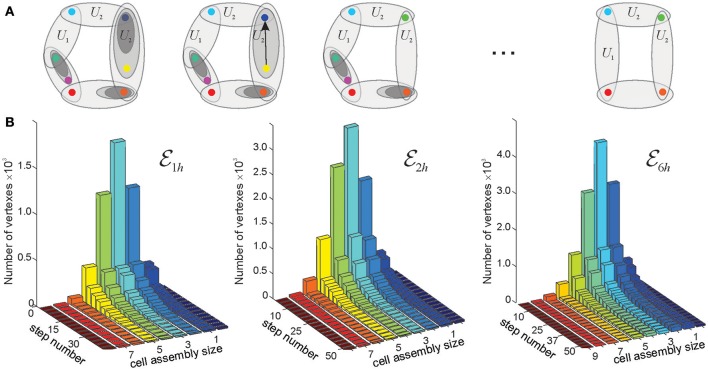
Reduction of finite topological spaces. **(A)** A finite topological space containing seven points, with the topological neighborhoods shown by the ovals. Note, that the blue and the yellow point on the first panel are separated by a single neighborhood. If this neighborhood is removed (middle panel), then the yellow point collapses onto the blue point. The resulting green point represents a single “combined” location. The minimal possible topological construction with circular topology contains four points. **(B)** The reduction of the number of points in three memory spaces, $M_{1h}\left(M\right)$, $M_{2h}\left(M\right)$, and $M_{6h}\left(M\right)$, in the three environments shown on Figure 3, as a function of the reduction step. As the topology is consolidated, the number of simplexes–and of the corresponding points–drops from thousands to a few dozens (see Figure 4). Note that the dimensionality of the original simplexes ranges between *D* = 7 for $T_{1h}$ and *D* = 9 for $T_{6h}$, whereas most elements in the reduced spaces have dimensionality *D* ≈ 3. Thus, the higher order memory combinations are consolidated into smaller-dimensional framework.

As mentioned in the section 2, topological neighborhoods define proximity and remoteness between spatial locations. However, certain neighborhoods may carry only limited topological information. For example, if a neighborhood *U*_*i*_ in a space A is entirely contained in a single larger neighborhood *U*_*k*_ and is involved in the same relationships with other neighborhoods as *U*_*k*_, then it only adds granularity to the topology of A without affecting its overall structure (Figure 5). In such case, the topology Ω(A) can be coarsened by removing *U*_*i*_ and producing a “reduced” space $A^{\prime }$ that is topologically similar to A (homotopically equivalent, see section 4 and McCord, 1966; Stong, 1966; Osaki, 1999). If such coarsening procedure is applied multiple times, then the resulting chain of transformations, $A\to A^{\prime }\to A^{\prime \prime }\to \ldots \to A^{\left(n\right)}$, generates a sequence of progressively coarser and coarser spaces that retain the homological identity of A (e.g., same Betti numbers).

**Figure 6:**
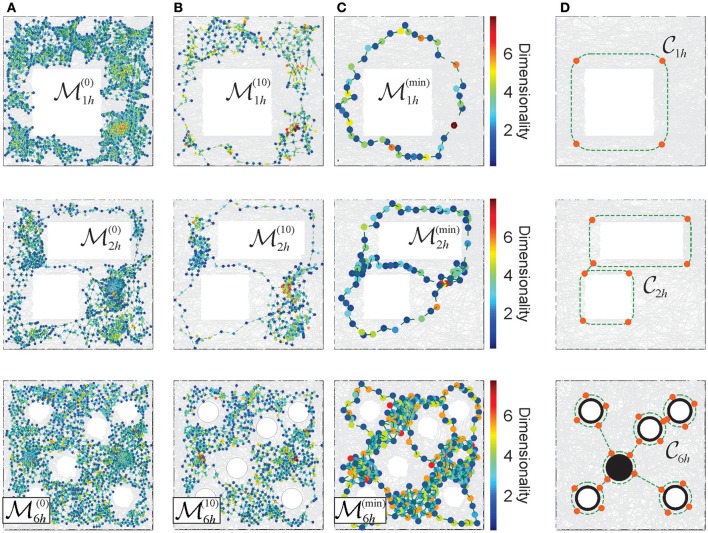
Reduction of the Alexandrov spaces into their cores and the corresponding Morris' schemas. **(A)** Points of the memory space, induced from a cell assembly coactivity complex, constructed for the place field maps shown on Figure 3. The color of points corresponds to the dimensionality of the corresponding simplexes. **(B)** The points of the memory spaces after 10 reduction steps (left column) and **(C)** the points of the topological cores of the memory spaces, obtained after a maximal reduction of the memory spaces (right column). For more examples see Supplementary Figure 1. **(D)** The minimal cores that correspond to each environment. A four point core on the top panel provides a minimal topological representation of a circle, the two linked four point circles represent the environment with two holes (middle panel). In the case of the environment considered in the experiment discussed in Tse et al. (2007), Figure 3D, the minimal core corresponds to the Morris' schema.

To the extent to which the consolidated memory frameworks retain the structure of the memory space $M_{E}$, they can be interpreted as its topological reductions. Thus, in the proposed approach, the consolidation process may be modeled via a sequence of less granular and more compact memory spaces, $M_{E}\to M_{E}^{\prime }\to M_{E}^{\prime \prime }\to \ldots \to M_{E}^{\left(n\right)}$ as discussed in McCord (1966), Stong (1966), and Osaki (1999), see Figures 6A–C and Supplementary Movies 4–6).

Importantly, the reduced memory spaces $M_{E}^{\left(k\right)}$ remain continuous images of both the original memory space $M_{E}$ and of the environment E. However, unlike the full memory space, the reduced memory spaces are not just “topological replicas” of the cell assembly complex: as the memory space is reduced, the direct correspondence between the simplexes of $T_{CA}$ and the elements of $M_{E}^{\left(k>0\right)}$ disappears. The reduction of neighborhoods and points in $M_{E}^{\left(k>0\right)}$ corresponds to elimination of certain simplexes of the cell assembly complex $T_{CA}$, i.e., to a restriction of the processed place cell coactivity inputs. The connections required to process these inputs can form a smaller cell assembly network that encodes the consolidated memory space $M_{E}^{\left(k\right)}$.

The smallest memory space obtained at the last step of the reduction process $M_{E}^{\left(\max\right)}$ (i.e., the one that cannot be reduced any further), retains the overall topological properties of the original memory space in the most compact form, i.e., using the smallest number of points and neighborhoods obtainable via a particular consolidation process (Figure 6C). The exact structure of such an “irreducible” memory space, referred to as a *core* $C(M_{E})$ of the memory space $M_{E}$, depends on the reduction sequence (McCord, 1966; Stong, 1966; Osaki, 1999 and Supplementary Figure 1). However, for every environment E, considered as topological space, there exists a unique core $C_{E}$ (see Figure 6D and Stong, 1966; Osaki, 1999), which schematically represents its basic, skeletal structure, approximated by $C(M_{E})$.

Similar compact, schematic representations of the memory structures are frequently discussed in neurophysiological literature. For example, in Tse et al. (2007) it was proposed that, as a result of learning, animals may acquire a “cognitive schema”—a consolidated representation of the spatial structure of the environmental and of the behavioral task (Morris, 2006; Tse et al., 2008). Specifically, in the case of the environment $E_{6h}$ shown on Figure 3C, the Morris' schema has the form shown on the bottom panel of Figure 6D, i.e., it is structurally identical to the core of $E_{6h}$. We use this observation to suggest that the Morris' schemas may in general be identified with the cores of the memory spaces produced by a particular cell assembly network in a given environment, and that acquiring a Morris' schema through a memory consolidation process may be modeled as the memory space reduction.

Under such hypothesis, the model allows computing specific Morris' schemas from their respective memory spaces, using the physiological parameters of neuronal activity and the corresponding cell assembly network architecture. Specifically, one can identify the number of elements in a given schema, their projected locations in the environment and their shapes. For the memory spaces constructed for different place field maps of the environments shown on Figure 3, the computed Morris schemas form a set of connected loops encircling the topological obstacles, as suggested in Tse et al. (2008) and Morris (2006). The density of the nodes along the constructed Morris' schemas (Figure 6C) is higher than in heuristic constructions, and similar to the characteristic distance between the place field centers in the corresponding maps.

## 4. Discussion

According to the cognitive map concept, spatial cognition is based on internalized representation of space encoded by the hippocampal network (Tolman, 1948), which was broadly studied both experimentally and theoretically, in particular, using the topological approach (Curto and Itskov, 2008; Chen et al., 2012; Dabaghian et al., 2012; Babichev et al., 2016b). Here we extend the topological schema approach proposed in Babichev et al. (2016a), to describe not only spatial, but also non-spatial memories in a single mathematical construct—a topological space with specific mathematical properties, induced by the physiological parameters of neuronal activity. The resulting model allows demonstrating, first, that the memory spaces incorporate representations of spatial experiences, i.e., that the cognitive maps are naturally embedded into memory spaces. In particular, the latter captures the topological structure of the navigated environment, so that the physical trajectories are represented by continuous paths in the memory space. Second, the model allows interpreting the hippocampal remapping phenomena in the context of the net topological properties of the memory spaces, both from the algebraic and from the general topological perspectives. Lastly, it connects the memory space structure to the Morris' schemas, by providing a schematic representation for the memory consolidation process.

### 4.1. Memory spaces in other topological schemas

Simplicial coactivity complexes, e.g., the ones discussed in the Examples 2 and 3 of section 2, are used to represent spatial information by a population of readout neurons responding to nearly simultaneous activity of the presynaptic place cells (Babichev et al., 2016a). However, the construction of the memory space discussed above is by no means limited to the particular syntax of processing the spiking outputs of the place cells. The key property of a simplicial complex that turns it into a space is the partial ordering of its simplexes, produced by the containment relationship: σ_1_ is “smaller” than σ_2_, if σ_2_ contains σ_1_ (i.e., σ_1_ < σ_2_ if σ_2_ ∩ σ_1_ = σ_1_). However, all topological schemas discussed in Babichev et al. (2016a) define partial orders, and without going into mathematical details, we point out that all partially ordered sets—*posets*—can be viewed as topological spaces, regardless of the nature of the order relationships (Vickers, 1989; Davey and Priestley, 2002). Thus, each topological schema S defines a specific finitary topological space, $M_{S}$, which can be interpreted as the memory space encoded by the cell assembly network that S represents. For example, a mereological schema F, based on the cover relation, defines partial order “covered region *x* is smaller than the covering region,” (*x* < *y* iff *x* ◂ *y*). The RCC5 schema $R_{5}$, based on five topological relations (partial overlap PO, proper part PP, its inverse PPi, discrete DR and equal EQ, see Figure 1A and Cui et al., 1993; Cohn et al., 1997) is also partially ordered. In this case, a region *x* is smaller than *y* if *x* is a proper part of *y*, or, if two regions *x* and *y* partially overlap, PO(*x, y*), then they share a *smaller* region *z* that is a proper part of both *x* and *y*, i.e., PP(*z, x*), PP(*z, y*) (Renz, 2002). The discrete (DR) or equal (EQ) regions are unrelated. The posets $P_{F}$ and $P_{R}$ corresponding to these schemas define their respective finitary topological spaces $M_{F}$ and $M_{R}$ that represent the topology of the environment just as the simplicial schema $M_{T}$ discussed above.

Given the same physiological parameters (e.g., the same number of place cells) the memory spaces produced by different schemas may differ from one another, e.g. some of them may have stronger topologies than others. However, all memory spaces may be regarded as finitary topological spaces and hence can be considered on the same footing, irrespective of the specific set of rules according to which the information provided by individual place cells is combined in S. Thus, the proposed model of memory spaces allows relating the capacity of different cell assembly networks, which may potentially implement different computational principles for processing and representing information.

### 4.2. Intrinsic representation of space

Current understanding of hippocampal neurophysiology rests on the assumption that place cells' spiking “tags” cognitive regions. Such approach allows describing the information contained in the spike trains phenomenologically, without addressing the “hard problem” of how the brain can intrinsically interpret spiking activity as “spatial” (Chalmers, 1995). It therefore remains unclear in what sense the spiking activity may actually produce a “cognitive region,” in what sense two such regions may “overlap” or “contain one another,” and so forth. Yet, in neuroscience literature it is recognized that “*allocentric space is constructed in the brain rather than perceived, and the hippocampus is central to this construction*” (O'Keefe and Nadel, 1978; Nadel and Eichenbaum, 1999). Paraphrasing Nadel and Eichenbaum (1999), it remains unclear how can “spaceless” data enter the hippocampal system and spatial cognitive maps come out. In this connection, we would like to point out that the topological approach discussed above may shed light on this problem, by allowing to interpret spatiality in purely relational terms, as a construct emerging from the relationships between the signals, implemented by neuronal networks with specific architecture.

## Mathematical and computational methods

Establishing a topological correspondence between the environment and the memory space requires a few definitions.

**Figure 7:**
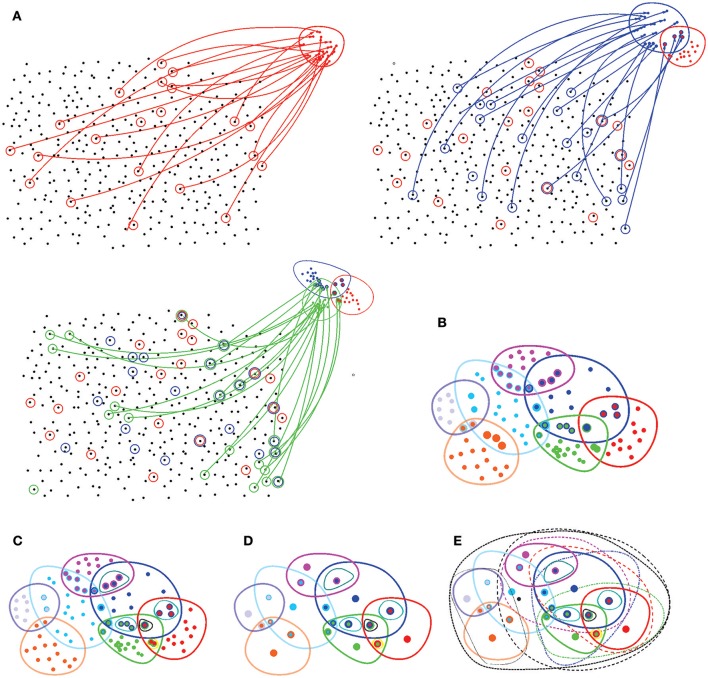
Basic notions of point set topology. **(A)** A set *X* with no spatial structure turns into a topological space as its elements are combined into topological neighborhoods. For example, a subcollection of elements of *X* (marked by red circles) may be selected to form the neighborhood “red” points, *U*_*r*_. Another collection of elements (blue circles) may form another, “blue” neighborhood *U*_*b*_ that may overlap with the red neighborhood *U*_*r*_, yet another set may form the green neighborhood *U*_*g*_, and so forth. **(B)** Eventually, the elements of *X* are grouped into a system of neighborhoods—in this case, seven neighborhoods. **(C)** All intersections between these neighborhoods define a topological base 𝔅—a set of basic neighborhoods whose combinations yield arbitrary neighborhoods on *X*. **(D)** The topology base defines the “resolution” of the corresponding topology: if two points share an identical system of neighborhoods, then they cannot be separated from each other, or “resolved” by the corresponding topology. The spaces in which for every two points *x* and *y* there is a neighborhood that contains one point but not the other are referred to as *T*_0_ spaces. In particular, all Alexandrov spaces are *T*_0_-separable. In the illustrated example, the topology base can “resolve” only 20 points, whereas all other elements of *X* fuse into these representative “locations.” **(E)** Adding the unions (only some unions are illustrated by black dashed lines) produces the full system of neighborhoods, a finitary topology Ω(*X*).

1. A *topology* on a space *X* is established by a system Ω(*X*) of topological neighborhoods, which obey the Hausdorff axioms: any unions and finite overlaps of the topological neighborhoods *U*_*i*_ ∈ Ω(*X*) produce another neighborhood from the same system Ω(*X*) (Figure 7). The empty set and the full set *X* also belong to Ω(*X*) (Alexandrov, 1965).

2. A *topology base* 𝔅 = *B*_*i*_ consists of a smaller set of “base” neighborhoods that can be combined to produce any other neighborhood *U*_*i*_ of Ω. A key property of a topology base is that it is closed under the overlap operation: an intersection of any two base neighborhoods yields (or, more generally, contains) another base neighborhood. A topology base generates a unique topology for which it forms a base, and hence it is a convenient tool for studying topological spaces (a rough analogy is a set of basis vectors in a linear space, see Alexandrov, 1965).

*Example 1: Euclidean plane*. The standard choice of a topological base 𝔅_*E*_ of a Euclidean domain E are the open balls of rational radii, centered at the points with rational coordinates. Every non-empty overlap of a finite collection of such balls contains a ball with a smaller radius. The full set of the topological neighborhoods in the resulting topology is given by the arbitrary unions of these balls (Alexandrov, 1965).

**Figure 8:**
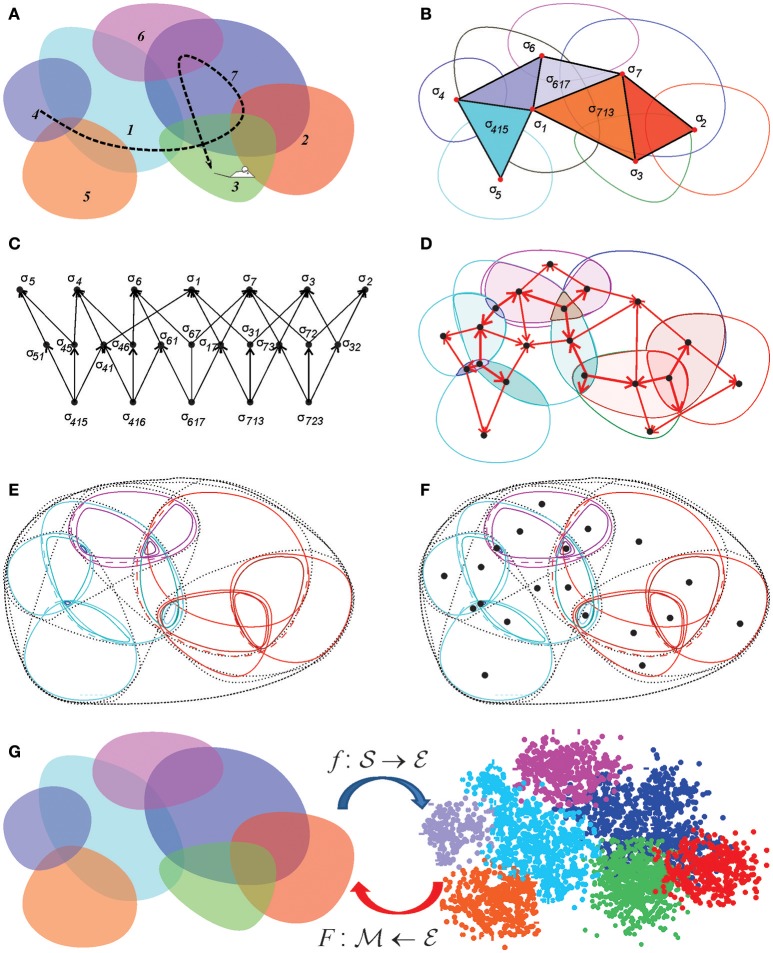
Discrete topological spaces and place field maps. **(A)** A schematic, mini-place field map that consists of seven place fields (colored ovaloids), traversed by a fragment of the animal's trajectory (dashed line). **(B)** The corresponding nerve complex $N_{7}$, which contains topological information about the environment. Its vertexes, σ_*i*_, correspond to the place fields, links σ_*ij*_, to overlapping pairs, the triangles σ_*ijk*_ to simultaneously overlapping triples of place fields. Alternatively, one can view this as the coactivity complex $T_{7}$, whose vertexes correspond to active place fields, links to pairs of coactive cells, triangles to coactive triples of cells, etc. **(C)** The partially ordered set–*poset* $P_{7}$ corresponding to the nerve $N_{7}$. **(D)** The simplexes of the simplicial complex $N_{7}$ (or the elements of the poset $P_{7}$) map into the atomic elements of the place field map. **(E)** The poset $P_{7}$ can be viewed as a pointfree (relational) space built from the regions defined by the place cell (co)activity. **(F)** The corresponding point-based Alexandrov space should be viewed as an analog of Figure 1B. **(G)** A spatial mapping from the memory map to the environment and the continuous mapping from the environment into memory space, $M_{T}$.

*Example 2: Cover induced topologies*. One can generate an alternative topology for the Euclidean domain E by covering it by a set of regions *U*_*i*_ and by augmenting this set with the regions obtained by all possible intersections *U*_*i*_ ∩ *U*_*j*_ ∩ …∩ *U*_*k*_. By construction, the resulting system of regions will be closed under the overlap operation and hence define a topology base 𝔅_*U*_. To obtain a topological base that is as rich as the Euclidean base 𝔅_*E*_, the collection of cover regions should be sufficiently large (certainly infinite). However, one can generate much more modest bases and topologies using finite covers. In particular, one can construct a topology of the environment starting from the place fields covering the environment E,

(1) $$E=\cup_{c=1}^{N_{c}}\pi_{c}$$

and build a discrete approximation to the Euclidean topology base from the place field domains and their intersection closure (Figures 3, 8).

*Example 3: Alexandrov topology on a simplicial complex*. In a simplicial complex Σ, a neighborhood *U*_σ_ of a simplex σ is formed by the set of simplexes σ_*m*_, *m* = 1, …, *n*_σ_, that include σ (Figure 2A). It can be verified directly that the unions and the intersections of so-defined neighborhoods generate other neighborhoods from Ω(A(Σ)), in accordance with the Hausdorff axioms (Alexandroff, 1937). The overlap of all the neighborhoods containing a given simplex σ, *U*_σ_ = ∩_*m*_*U*_σ_*m*__, is its minimal neighborhood. The minimal neighborhoods form a topology base in finitary space $A_{\Sigma }$, which defines the Alexandrov topology Ω(A(Σ)) (Figure 8). In particular, the Alexandrov topology is defined for all the examples discussed in section 2: the nerve complex N, the temporal complex T and the cell assembly complex $T_{CA}$.

### 4.3. Continuous mappings between topological spaces

A space *X* maps continuously onto a space *Y*, *f* : *X* → *Y*, if each topological neighborhood in *Y* is an *f*-image of a topological neighborhood in *X* (for precise discussions see Munkres, 2000). If two spaces *X* and *Y* map continuously onto each other, then they are topologically equivalent. An example of topological equivalence is a continuous deformation of *X* into *Y* (one can imagine the corresponding deformation of the neighborhoods of *X* into the neighborhoods of *Y* that does not violate the mutual overlap, containment and adjacency relationships between the neighborhoods). In contrast, if *X* cannot be transformed into *Y* without adding or removing neighborhoods and points, then *X* and *Y* are topologically distinct. For example, if a space *Y* contains an extra hole, then the topology on *Y* lacks neighborhoods that relate the “missing” points (contents of the hole) and points outside of the hole. For this reason, a mismatch in the number of holes, handles, connectivity components and similar qualitative features serves as an immediate indicator of topological inequivalence of spaces.

It is important to notice, that if the space *X* has a richer topology (i.e., a larger set of topological neighborhoods) than *Y*, then a continuous mapping *f* : *X* → *Y* may exist, but an inverse mapping, *g* : *Y* → *X*, will not. For example, the rich Euclidean topology of the environment E can map continuously into the finitary topology of the memory space M, because many neighborhoods of E may map into a single neighborhood of M. The converse is not true: no mapping can reproduce the infinity of open sets in E from a finite set of neighborhoods in M.

### 4.4. A continuous mapping of the environment into the memory space

A continuous mapping of the environment into the memory space can be constructed as follows. Let us consider first the coactivity complex T and a spatial mapping, $f:M_{T}\to E$, that ascribes the Cartesian (*x, y*) coordinates to the spikes according to the animal's location at the time of spiking (Babichev et al., 2016a) (Figure 8G). This function maps the activity of an individual place cell into its place field, *f* : *r*_*i*_ → π_*i*_, and the firing pattern of a place cell combination σ into its simplex field *l*_σ_—the domain where all the cells in σ are active, *f*:σ → *l*_σ_. Notice that simplex fields exist for all (not only maximal) simplexes of T. If some combination of place cells is active at every location of the environment (a physiologically justified assumption), then the simplex fields form a cover of E,

(2) $$E=\cup_{\sigma =1}^{N_{\sigma }}l_{\sigma }$$

Since simplexes of T may overlap with or include one another, the corresponding simplex fields may also overlap. However, for every simplex σ there generically exists a subregion of its simplex field—the *atomic* region *a*_σ_—where *only* this specific combination of cells is active. The name “atomic” emphasizes that these regions cannot be subdivided any further based on the information provided by place cell coactivity (a non-empty overlap of *a*_σ_ with any other region yields *a*_σ_) and that they are disjoint ($a_{\sigma }\cap a_{\sigma }^{\prime }=\emptyset$ for σ ≠ σ′). As a result, they form a partition of the environment—the atomic decomposition of the cover:

(3) $$E={⊔}_{\sigma =1}^{N_{\sigma }}a_{\sigma }$$

which may be viewed as the ultimate discretization of space produced by the given place field map.

Since each atomic element corresponds to a particular simplex σ of T, it also defines a point *x*_σ_ of $A_{T}$, and hence an element of the memory space $M_{T}$. Consider now a reverse mapping, $F:E\to M_{T}$, in which every point *r* = (*x, y*) of the environment contained in the atomic region *a*_σ_ maps into the corresponding point *x*_σ_ of $M_{T}$. By construction, every base (minimal) neighborhood in memory space $\Omega \left(M_{T}\right)$ is an image of a base neighborhood in the Euclidean topology of the environment, Ω(E), and hence *F* is continuous map.

### 4.5. Continuity in memory space encoded by the cell assembly network

A similar argument applies to the memory space generated by the cell assembly complex $T_{CA}$. Similarly to the previous case, we assume that at least one cell assembly or its subassembly is active in every location of the environment (Babichev et al., 2016b) and hence that the place cell (sub)assembly fields 𝔩_σ_ form a cover

(4) $$E=\cup_{\sigma =1}^{N_{\sigma }}l_{\sigma }$$

The intersection closure of the cell assembly cover yields the decomposition of the environment into the non-overlapping atomic regions 𝔞_*k*_, which form a partition of the environment,

(5) $$E={⊔}_{k=1}^{N_{k}}a_{\sigma }$$

Since every point of the environment belongs to one atomic region that corresponds to a particular minimal neighborhood of the memory space, we have a continuous mapping from E to $T_{CA}$ and hence $M_{E}$.

Alternatively, one can establish continuity of E to $T_{CA}$ by constructing a simplicial mapping from the coactivity complex T to its subcomplex $T_{CA}$, based on the observation that both complexes are connected, have finite order, free fundamental groups and identical homologies (Babichev et al., 2016b).

### 4.6. Stong matrix

The numerical analyses of the finite memory spaces were carried out in terms of the Stong matrices. If a finite topological space *X* contains *N* minimal neighborhoods, *U*_1_, *U*_2_,…, *U*_*N*_, then the topological structure on *X* is uniquely defined by a matrix *M*_*ij*_, defined as following:

1. *M*_*ii*_ = number of points that fall inside of the neighborhood *U*_*i*_;
2. if *U*_*i*_ is the immediate neighborhood of *U*_*j*_, *M*_*ij*_ = 1 and *M*_*ji*_ = −1;
3. *M*_*ij*_ = 0 otherwise;

Conversely, every integer matrix satisfying the requirements 1-3 describes a finite topological space A (Stong, 1966).

For two finitary spaces A and B, topological equivalence follows from the equivalence of the corresponding Stong matrices: A is equivalent to B, if the topology Ω(A) can be obtained from Ω(B) by re-indexing the minimal neighborhoods. In other words, A and B are topologically equivalent if the Stong matrix $M_{A}$ can be obtained from the Stong matrix $M_{B}$ by a permutation of rows and columns, otherwise they are topologically distinct (Stong, 1966).

### 4.7. Reduction of a stong matrix

If minimal neighborhood *U*_*i*_ is contained in a single immediate neighborhood *U*_*k*_, then it only adds granularity to the Alexandrov space A. The latter can then be coarsened by removing *U*_*i*_. If, as a result of coarsening, the neighborhoods separating two points *p*_1_ and *p*_2_ disappear, then they fuse into a single point. This yields a “reduced” Alexandrov space $A^{\prime }\equiv A^{1}$ that is weakly homotopically equivalent to $A\equiv A^{0}$ (Stong, 1966; Osaki, 1999). Such coarsening procedure can be applied multiple times: the resulting chain of transformation of A can be viewed as a discrete homotopy process, $A^{\left(0\right)}\to A^{\left(1\right)}\to A^{\left(2\right)}\to \ldots \to A^{\left(n\right)}$, leading to more and more “coarse” topologies (Figure 3).

The numerical procedure implementing the Alexandrov space reduction is as follows. If a column *m*_*i*_ of a Stong matrix contains only one non-zero element *m*_*ik*_, it is removed along with the corresponding row, then the *n* × *n* matrix $M_{A}$ reduces to a (*n* − 1) × (*n* − 1) matrix $M_{A}^{\prime }$. Eventually, the Stong matrix reduces to a “core” form that cannot be reduced any further; the corresponding Alexandrov space $C_{A}$ is referred to as the core of the original Alexandrov space A. The reduction process is illustrated in Supplementary Movies 4–6.

### 4.8. Proximity between topologies

One can quantify difference between finite topologies Ω_1_ and Ω_2_ by estimating the norm of the difference between the corresponding Stong matrices *M*_1_ and *M*_2_, minimized over the set *P* of all row and column permutations,

(6) $$D_{P}(M_{1},M_{2})=\underset{P}{\min}|P(M_{1})-M_{2}|.$$

As a simpler option, one can evaluate the distance between the reduced row echelon forms of the Stong matrices,

(7) $$D(M_{1},M_{2})=|(rref(M_{1})-rref(M_{2})|,$$

illustrated Figure 4. Clearly, both distances Equations (6) and (7) vanish if the matrices *M*_1_ and *M*_2_ are equivalent, i.e., if the corresponding memory spaces are homeomorphic.

### 4.9. Computational algorithms

Computational algorithms used to simulate the place cell activity are outlined in Dabaghian et al. (2012), Arai et al. (2014),and Babichev et al. (2016b).

## Author contributions

YD conceived of the project, carried out computations, wrote the paper. AB carried out computations, produced materials for figures.

### Conflict of interest statement

The authors declare that the research was conducted in the absence of any commercial or financial relationships that could be construed as a potential conflict of interest.

## Supplementary material

Supplementary Figure 1 Cores of the memory spaces in three environments. The figure demonstrates cores of the memory spaces obtained for 10 different place field maps in the three environments shown on Figure 3. The shapes of the cores depend on the map and on the reduction sequence, however they capture the structure of the environment and approximate its topological core.

Supplementary Movie 1 **Left panel** shows navigation along a trajectory γ(*t*) in physical space with one hole (Figure 3A). The corresponding trajectory ℘(*t*) in memory space is shown on the **right panel**.

Supplementary Movie 2 Navigation in the memory space **(left panel)** and the corresponding activity in the simulated cell assembly network **(right panel)**.

Supplementary Movie 3 **Left panel** shows navigation in physical space with five holes discussed in (Tse et al., 2007) (see Figure 3C) represented by a trajectory in memory space **(right panel)**.

Supplementary Movie 4 Reduction of the memory spaces in the environments shown on Figure 3. At each reduction step multiple points are removed simultaneously.

Supplementary Movie 5 Same as previous file.

Supplementary Movie 6 Same as previous file.
