# Supplementary figures and images for: Topological Schemas of Memory Spaces
Source: Front Comput Neurosci. 2018 Apr 24;12:27. doi: 10.3389/fncom.2018.00027 (PMC5928258; doi:10.3389/fncom.2018.00027)

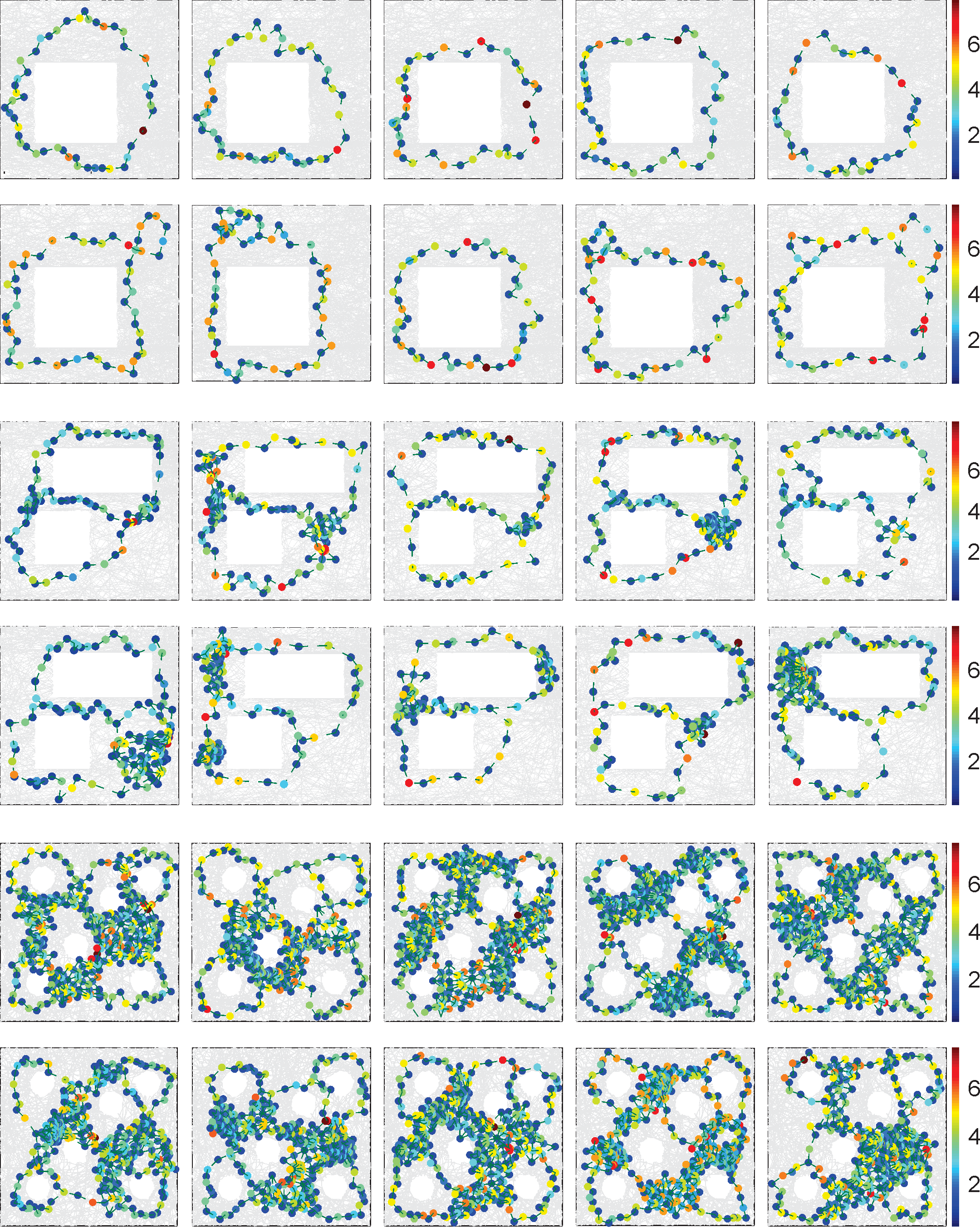

Supplement: Supplementary Figure 1 — Cores of the memory spaces in three environments. The figure demonstrates cores of the memory spaces obtained for 10 different place field maps in the three environments shown on Figure 3. The shapes of the cores depend on the map and on the reduction sequence, however they capture the structure of the environment and approximate its topological core. [file Image_1.tif]
